# Mapping the Human Exposome to Uncover the Causes of Breast Cancer

**DOI:** 10.3390/ijerph17010189

**Published:** 2019-12-27

**Authors:** Vincent Bessonneau, Ruthann A. Rudel

**Affiliations:** Silent Spring Institute, 320 Nevada Street Newton, Newton, MA 02460, USA; rudel@silentspring.org

**Keywords:** breast cancer, exposome, metabolomics, adductomics

## Abstract

Breast cancer is an important cause of morbidity and mortality for women, yet a significant proportion of variation in individual risk is unexplained. It is reasonable to infer that unexplained breast cancer risks are caused by a myriad of exposures and their interactions with genetic factors. Most epidemiological studies investigating environmental contribution to breast cancer risk have focused on a limited set of exposures and outcomes based on a priori knowledge. We hypothesize that by measuring a rich set of molecular information with omics (e.g., metabolomics and adductomics) and comparing these profiles using a case-control design we can pinpoint novel environmental risk factors. Specifically, exposome-wide association study approaches can be used to compare molecular profiles between controls and either breast cancer cases or participants with phenotypic measures associated with breast cancer (e.g., high breast density, chronic inflammation). Current challenges in annotating compound peaks from biological samples can be addressed by creating libraries of environmental chemicals that are breast cancer relevant using publicly available high throughput exposure and toxicity data, and by mass spectra fragmentation. This line of discovery and innovation will extend understanding of how environmental exposures interact with genetics to affect health, and provide evidence to support new breast cancer prevention strategies.

## 1. Introduction

Breast cancer is the most common cancer in women worldwide, and is a major cause of death for women in mid-life [1]. In fact, breast cancer in women ages 20–49 is six times more prevalent than any cancer in men [2]. Despite interest in identifying preventable causes of breast cancer, a significant proportion of variation in individual breast cancer risk is unexplained [3,4], and so new approaches to prevention are needed. 

Increasing evidence suggests that environmental factors, rather that inherited genetic factors, are the major causes of chronic diseases, including breast cancer. Studies of cohorts of 44,788 pairs of Western European monozygotic twins estimated that the genetic population attributable fraction for breast cancer was 31% (95% CI: 11–51%) [5,6]. In other words, 31% of breast cancer cases were attributed to inherited genetic factors in this population. 

While several environmental factors, including history of hormone replacement therapy, reproductive history, alcohol consumption, and physical activity are associated with breast cancer risk [7,8], risk ratios (RRs) or odds ratios (ORs) are modest (e.g., ORs range from 1 to 2). It is reasonable to infer that unexplained breast cancer risks are caused by a myriad of environmental exposures—the exposome—and their interactions with genetic factors. Here, the exposome is defined as the cumulative measure of environmental influences such as dietary factors, drugs, chemical pollutants, behavioral factors and socio-economic factors, and associated biological responses throughout the lifespan [9].

Animal studies and epidemiology suggest that environmental chemicals likely play diverse biological roles in the development of breast cancer, with some exposures important early in life and others important in mid- or late-life. For example, our recent review of biological evidence and epidemiologic studies found a higher risk of breast cancer for exposures to dioxins, air pollution, dichlorodiphenyltrichloroethane (DDT), and perflurooctanesulfonamide (PFOSA) during breast development, and for occupational exposures to solvents and other mammary carcinogens such as gasoline components [10]. 

However, most epidemiological studies investigating environmental contribution to breast cancer risk have focused on a limited set of exposures and outcomes based on a priori knowledge and, as a result, are likely to miss important relationships. In addition, these studies have almost exclusively assessed the risks of exposure to single chemicals. In order to reduce the burden of breast cancer, there is an urgent need to find the undiscovered environmental risk factors. One promising approach is to conduct exposome-wide association studies (EWAS) that measure a rich set of molecular information from both exogenous and endogenous sources in biospecimens and identify associations with breast cancer [11,12]. Systems-based approaches that utilize “omics” technologies are now emerging as powerful approaches for mapping the exposome in order to discover features associated with higher risk. 

## 2. Approaches and Methods

**Figure 1:**
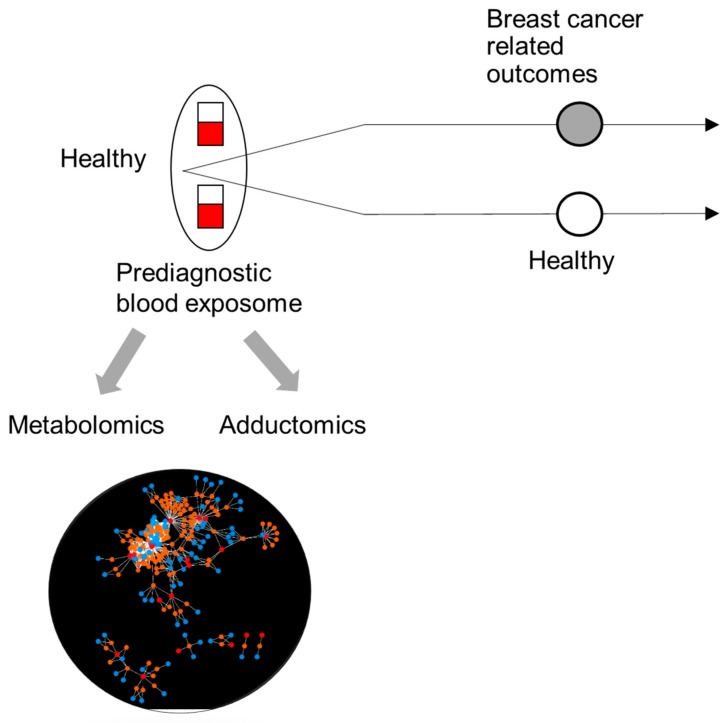
Conceptual diagram. Exposome-wide association studies using a prospective case-control design can discover novel environmental risk factors related to breast cancer. The exposome includes measurements of a rich set of molecular information in prediagnostic biospecimens. Cases can be breast cancer cases or cases with phenotypic measures related to breast cancer.

The objective of this research program is to measure a myriad of environmental exposures (i.e., the exposome), evaluate their interactions with genetic factors, and assess their associations with health outcomes and/or biological pathways related to breast cancer (e.g., chronic inflammation, breast density, and hormones levels) (Figure 1). We hypothesize that by measuring a rich set of molecular information (e.g., small molecules, protein, and DNA adducts) in archived biospecimens and using a case-control design where cases have breast cancer or defined by phenotypic measures associated with breast cancer (e.g., chronic inflammation and breast density), EWAS can pinpoint novel environmental risk factors associated with these outcomes [13,14,15]. This approach has been successfully used to discover novel risk factors for type 2 diabetes [16], blood pressure [17] and all-cause of mortality [18] in the U.S. Ideally, EWAS should be perform using a prospective study design with molecular information measured in prediagnostic biospecimens for establishing causality. 

### 2.1. Capturing Molecular Information Using Omics

Metabolomics is recognized as a powerful platform for detecting small molecules in biospecimens [19,20]. These small molecules can be either substrates or end products of cellular metabolism and can originate from exogenous sources via inhalation, ingestion and dermal absorption, or from endogenous processes including human and microbial metabolism. Very few metabolomics studies have explored associations of environmental exposures with the risk of breast cancer. A recent nested case-control study has evaluated associations between diet-related metabolites and postmenopausal breast cancer [21]. Among 113 prediagnostic diet-related metabolites—identified by computing partial correlations between serum metabolites and food items—three were associated with overall breast cancer risk (*N* = 621 cases) and nineteen with estrogen receptor positive breast cancer (*N* = 418 cases), including saturated fatty acids (from fats/oils), vitamin E derivatives (from desserts or vitamin supplements) and androgens (from alcohol), with ORs ranging from 0.6 to 2.2.

The main challenge with the use of non-targeted metabolomics based on high-resolution mass spectrometry (HRMS) is the annotation (i.e., identification) of small molecules. The first step for the annotation of metabolomics features relies on matching accurate mass of detected features to known small molecules present in public databases such as the Human Metabolome Database (HMDB) [22] or the METLIN database [23]. These databases contain information about more than 100,000 endogenous and exogenous metabolites, but very few entries are related to environmental chemicals. Therefore, metabolite databases must be extended by creating databases of environmental chemicals.

Data resources that are now available from the US Environmental Protection Agency (EPA) and other sources provide valuable information to help build environmental chemical databases that are relevant to breast cancer. To start, very large exogenous chemical libraries are available such as the EPA’s Distributed Structure-Searchable Toxicity (DSSTOX; ~875,000 chemicals) [24] or PubChem (6.5 million unique chemical structures) [25]. While these provide coverage of the chemical space, they contain so many chemicals that they may not be helpful in targeting chemicals that are ongoing exposures or that are plausible breast carcinogens, and there will be many chemicals matching to the mass of each observed peak. Thus, new approaches are needed to narrow these large databases to focus on chemicals with likely exposures and likely relevant biological effects. For example, we recently applied this approach to identify novel chemical exposures in a cohort of California women firefighters and office workers. Metabolomics analysis of serum samples showed tentative matches to more than 600 chemicals from our in-house database of 740 slightly polar and acidic compounds expected to have widespread exposures [26]. 

Additional opportunities to develop relevant chemical databases for annotating tentative chemical matches would use available data from EPA on expected population exposures and mechanisms of toxicity. For example, EPA has used high throughput modeling along with information on production volumes, chemical properties, and some chemical use information to predict population exposures for almost 500,000 chemicals. These data may be used to prioritize chemicals for a database. For example EPA’s modeling predicted 1880 chemicals would have median population exposures above 0.1 mg/kg-day [27]. Chemical databases should also be developed that feature chemicals shown to be active in breast cancer-related pathways, such as estrogen receptor activation or DNA damage. These chemicals can also be extracted from EPA’s ToxCast data, which includes results from over 700 different in vitro activity assays for 3000 chemicals [24]. 

Another challenge with annotation of metabolomics features is that it is often not possible to identify compounds simply based on accurate mass since several molecules with different structures exhibit similar mass. Therefore, successful studies should utilize HRMS to produce MS/MS fragmentation spectra. The generated MS/MS spectra will provide structural information specific to each molecule to guide identification of compounds. 

It is also important to note that non-targeted metabolomics analysis using HRMS is less sensitive, compared to targeted HRMS analysis, and therefore exposures to very low levels of environmental factors may be missed. New tools and approaches to improve the sensitivity of non-targeted HRMS metabolomics analysis would be greatly encouraged. 

Adductomics is another top-down technique that measures modifications of blood proteins like hemoglobin (Hb) or human serum albumin (HSA) to characterize exposures to reactive electrophiles that are inherently toxic but cannot be measured directly in biospecimens [11]. By characterizing a complete adductome—via non-targeted HRMS analysis of adducts of DNA, HSA, and Hb—it is possible to map systemic exposures that occurred over the in vivo residence time of the nucleophile (i.e., blood protein), which can range from hours to months [15,28]. This approach has been recently used to identify protein modifications of HSA in serum samples from Chinese workers exposed to benzene [29] as well as adducts produced by microbial metabolites and their relationships with colorectal cancer in a case-control study of Italian [30]. 

The integration of data from multiple omics platforms would provide a more holistic understanding of gene-environment interactions in breast cancer pathogenesis compared to single omics approaches analyzed in isolation. However, multi-omics integration faces several challenges such as data harmonization, large number of false positive results, inadequate data visualization tools, and metabolic pathway databases. 

### 2.2. Resources Needed

This research program would need to be carried out by a multidisciplinary team with expertise in omics measurements, bioinformatics, breast cancer biology, toxicology (including computational toxicology), and epidemiology. EPA data on short term toxicity testing and exposure modeling are publicly available and can be used to build libraries of breast cancer-relevant chemicals. The proposed research could leverage existing cohorts, which have archived biological specimens and have collected extensive health information, including known risk factors for breast cancer and breast cancer outcomes or phenotypes relevant to breast cancer. California is well positioned to carry out this research idea, with several state-of-the-art omics platforms, researchers with relevant expertise, and access to existing cohorts of adult women and young girls with archived biological specimens suitable for omics and information on breast cancer risk factors (e.g., the Child Health and Development Studies CHDS, Avon Army of Women, and the Sister Study). Projects designed to meet these objectives will need budgets in the range of $1 M/year for 4–5 years.

## 3. Expected Results and Discussion

We anticipate that this research program will build and demonstrate a new approach combining omics and phenotypic measurements to investigate associations of an unprecedentedly wide spectrum of exposures (i.e., exogenous and endogenous) with phenotypic measures, which will provide unique opportunities to discover novel environmental risk factors for breast cancer and bring new insights into molecular mechanisms of these relationships. Two key innovative elements are application and integration of omics approaches to identify differences between participants with and without breast cancer or breast cancer risk factors, and the creation of a new breast cancer-related chemical library of pharmaceuticals, food and consumer product chemicals, and pollutants that have biological activities similar to known breast carcinogens. The use of MS/MS fragmentation profiles will provide critical structural information to get more confident identification of compounds. Use of newly available databases of short-term toxicity and exposure predictions for thousands of chemicals to identify breast cancer-related chemicals is also novel as an approach for building a metabolomics compound database. 

Findings from this program will provide a newly extended list of breast cancer-related environmental factors and strengthen scientific support for reducing exposure to such factors by providing novel data about human exposures from diet, lifestyle factors, and chemicals that are associated with health outcomes and/or pathways related to breast cancer. We also expect that this program will identify endogenous mediators of the relationships between exposures and breast cancer-related phenotypes, providing new insights about molecular mechanisms underlying exposure–outcome relationships. This will further our understanding of how environmental exposures interact with genetics and impact women’s health, and will ultimately help develop prevention strategies and lower the incidence of breast cancer. Projects that would stratify women based on menopausal status, race/ethnicity and socio-economic level would provide an opportunity to also evaluate disparities in estimated breast cancer risk from exposure to environmental factors and identify disproportionately affected populations.

## 4. Conclusions

We hypothesize that by measuring a rich set of molecular information with omics (e.g., metabolomics and adductomics) and comparing these profiles using a case-control design we can pinpoint novel environmental risk factors for breast cancer. Specifically, exposome-wide association study (EWAS) approaches can be used to compare molecular profiles between controls and either breast cancer cases or participants with phenotypic measures associated with breast cancer (e.g., high breast density and chronic inflammation). Current challenges in annotating compound peaks from biological samples can be addressed by creating libraries of environmental chemicals that are breast cancer relevant using publicly available high throughput exposure and toxicity data, and by mass spectra fragmentation. This line of discovery and innovation will extend understanding of how environmental exposures interact with genetics to affect health, and provide evidence to support new breast cancer prevention strategies.
